# *Giardia* VSPAS7 protein attenuates *Giardia intestinalis*-induced host macrophage pyroptosis

**DOI:** 10.1186/s13071-023-05949-0

**Published:** 2023-10-11

**Authors:** Min Sun, Zhiteng Zhao, Ying Li, Lili Cao, Jianhua Li, Xichen Zhang, Xin Li, Nan Zhang, Shuqin Cheng, Xiaocen Wang, Pengtao Gong

**Affiliations:** 1 State Key Laboratory for Zoonotic Diseases, Key Laboratory for Zoonosis Research of the Ministry of Education, Institute of Zoonosis, and College of Veterinary Medicine, Jilin University, Changchun, 130062 China; 2 Jilin Academy of Animal Husbandry and Veterinary Medicine, Changchun, 130062 China

**Keywords:** *Giardia intestinalis*, VSPAS7, NLRP3, Pyroptosis

## Abstract

**Background:**

The unicellular protozoan parasite *Giardia intestinalis*, which primarily infects humans and animals such as cattle and sheep, is having a major negative impact on public health. *Giardia* is able to evade the recognition and elimination of the host immune system because of the trophozoite surface and extracellular vesicles (EVs) covered by variant-specific surface proteins (VSPs). As key proteins for immune evasion, whether VSPs can regulate *Giardia*-induced pyroptosis and promote *Giardia* evasion of host immune responses has not been reported.

**Methods:**

To examine the role of *Giardia* VSPAS7 on *Giardia*-induced activation of the signaling pathway, secretion of pro-inflammatory cytokines, pyroptosis and the mechanism involved, we constructed the pcDNA3.1-*vspas7* expression plasmid and transfected this plasmid into mouse macrophages. Key proteins for pyroptosis, IL-1β secretion and LDH release were detected in pcDNA3.1-*vspas7*-transfected wild-type (WT) cells and NLRP3-deficient cells by western blot, ELISA and LDH assays, respectively. The interactions of *Giardia* VSPAS7 and mouse NLRP3 were examined using immunofluorescence assays (IFA), co-immunoprecipitation (Co-IP) and bimolecular fluorescence complementation (BiFC) assays.

**Results:**

VSPAS7 could decrease the levels of phosphorylated-p65 (P-p65), P-IκBα and P-ERK caused by *Giardia* and reduce the production levels of *Giardia*-induced pro-inflammatory cytokine IL-6, IL-12 p40 and TNF-α. The results showed that VSPAS7 inhibited *Giardia*-mediated activation of NF-κB, ERK/MAPK signaling and secretion of pro-inflammatory cytokines. Furthermore, VSPAS7 suppressed *Giardia*-induced macrophage pyroptosis by reducing GSDMD cleavage, caspase-1 activation, IL-1β secretion and LDH release. We further found that VSPAS7 could interact with mouse NLRP3 directly, and in NLRP3-deficient cells the suppression of *Giardia*-induced macrophage pyroptosis by VSPAS7 was significantly attenuated.

**Conclusions:**

Overall, VSPAS7 could inhibit *Giardia*-induced activation of signaling pathways and pyroptosis in host macrophages, allowing *Giardia* evasion of host immune responses. Studies on *Giardia* VSP-mediated immune evasion provide an important theoretical basis for in-depth studies on *Giardia* pathogenicity.

**Graphical abstract:**

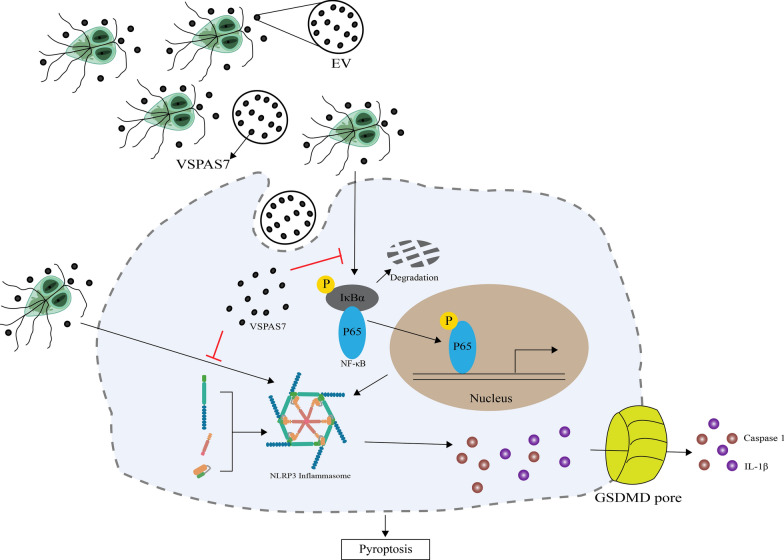

**Supplementary Information:**

The online version contains supplementary material available at 10.1186/s13071-023-05949-0.

## Background

*Giardia intestinalis*, also known as *G. lamblia* and *G. duodenalis*, is one of the most common intestinal parasites in humans and animals such as sheep and cattle, with the major symptoms being diarrhea and malabsorption [1]. Approximately 200 million individuals worldwide are infected with *G. duodenalis* [2], and this infection is mostly self-limiting and inapparent, but it also frequently recurs and becomes chronic [3]. It can be chronic and recurrent for a variety of reasons, mainly including altering variant-specific proteins (VSPs) [4] on the parasite surface to avoid detection and clearance by the host cells [5].

Variant-specific surface proteins cover *Giardia*'s trophozoite surface (including flagella and ventral disc) and belong to a family of related and highly unusual proteins. There are approximately 200 VSPs in *Giardia* [4], but just one VSP is ever expressed at any one time on a single trophozoite surface [6], while multiple VSPs are expressed simultaneously during cyst formation [7]. This protein family is typically characterized by a varied N-terminal region, a conserved transmembrane region and a CRGKA intracellular tail [8]. Every 6–13 generations, each VSP on the trophozoite's surface undergoes a spontaneous conversion, which may be a self-defense mechanism used by *Giardia* to prevent being identified and removed by the immunological system of the host [9]. In addition, VSP1267, VSP9B10 and VSPH7 have been found to activate the host innate immune responses [10]. Interestingly, Zhao et al. found that proteomic analysis of extracellular vesicles (EVs) of *G. duodenalis* showed that VSPs were also present in EVs [11]. Although VSPs have the dual function of mediating the activation of the host autoimmune responses by *Giardia* and assisting immune evasion [12], it is not known whether there are other ways in which VSPs can modulate the innate immunological response of the host.

It has been reported that *Giardia* can mediate peritoneal macrophage pyroptosis via NLRP3/caspase-1/GSDMD in mice [13], contributing to the host resistance to *Giardia* infection and pathogenesis. Pyroptosis, a controlled cell death that is a component of the host's innate immune responses, is dependent on the creation of plasma membrane holes by proteins from the gasdermin family [14]. Cell swelling, plasma membrane rupture, chromatin breakage and the release of inner pro-inflammatory substances are its distinguishing features [15, 16]. There are three pyroptosis pathways: the classical [17–20], the non-classical [21–23] and the novel pathway [24–27]. The classical pathway is the most widely studied and consists of three components: pattern recognition receptor (PRR) proteins, the linker protein ASC and caspase-1 [18]. When PRR proteins recognize pathogen-associated molecular patterns or damage-associated molecular patterns (PAMPs and DAMPs), the recognition signal is transmitted to the junction protein ASC, which recruits intracellular caspase-1 precursors to bind and form inflammasomes [19]. Subsequently, the inflammasome complexes undergo oligomerization, bringing about the activation of caspase-1 precursors to enzymatically active caspase-1, which induces the cleavage of gasdermin D (GSDMD) and the release of its N-terminal fragment, forming a non-selective pore in the cell membrane. Activated caspase-1 also cleaves IL-1β and IL-18 into their mature forms [28], resulting in cell swelling and pyroptosis [15, 20]. It has been shown that *Giardia* can activate host macrophage pyroptosis and *Giardia*-secreted protein PPIB [13] can also induce host pyroptosis. Interestingly, VSPs, such as VSPAS7, were also detected in *Giardia* EVs [11]. *Giardia* EVs could enter the host cell via endocytosis and mediate the activation of NLRP3 inflammasome, which next triggers the host inflammatory response [11, 29]. However, it has not been reported whether VSPs can also regulate host pyroptosis.

In this study, we selected VSPAS7 to see if it could facilitate immune evasion by regulating *Giardia*-induced host pyroptosis. VSPAS7 was chosen from the repertoire of *Giardia*’s VSPs found in EVs [11]. We analyzed the role of VSPAS7 in *Giardia*-mediated pyroptosis in mouse primary macrophages in vitro and sought to identify the inflammasomes involved in this procedure. We found that *Giardia* VSPAS7 regulated *Giardia*-induced host macrophage pyroptosis, and further studies determined that this process was accomplished through NLRP3 inflammasome.

## Methods

### Ethics statement

All animal experiments were conducted in strict accordance with the Regulations for the Administration of Affairs Concerning Experimental Animals approved by the State Council of the People's Republic of China (1988.11.1) and the Animal Welfare and Research Ethics Committee of Jilin University (IACUC permit number: 20160612).

### Plasmid construction

Based on the GL50803_101496 gene (*vspas7*) in GiardiaDB [5], its seamless cloning primers were 5'-TGGTGGAATTCTGCAGATATGGCCTACAAGAGTGCACCC-3' and 5'-GCCGCCACTGTGCTGGATACGCCTTACCCCGGCAGAGGA-3'. The N-terminal signal peptide of VSPAS7 is absent. At the C-terminus, there is a transmembrane domain. The nature stop codon was removed by primer design. At the protein's N- or C-terminus, no epitope tags were inserted. The pcDNA3.1 vector contained the His tag that was utilized for the experiment. The target gene was obtained after PCR amplification. Afterwards, the pcDNA3.1 vector was digested using EcoRV restriction endonuclease. The amplified gene was ligated to the vector to construct the pcDNA3.1-*vspas7* recombinant vector.

### Isolation and cultivation of mouse peritoneal macrophages

The female C57BL/6 mice were purchased from Changsheng Laboratory Animal Centre (Anshan, China). The separation and cultivation protocol of mouse peritoneal macrophages (PMs) was based on the method from Pu et al. [30]. Briefly, mouse primary PMs were enriched by intraperitoneal injection of 2–3 ml of 2.98% DIFCO fluid thioglycollate medium (Becton, Dickinson and Company, Sparks, MD, USA) into C57BL/6 mice. Four days later, PMs were harvested. After counting, 4.5 × 10^6^ cells per well were cultured at 37 °C and 5% CO_2_ for 6 h, and then fresh RPMI1640 medium with 10% fetal bovine serum, 100 U/ml penicillin and 100 mg/ml streptomycin (Biological Industries) was added prior to further use.

### Eukaryotic vector transfection

The pcDNA3.1-*vspas7* expression vector was transfected using Lipofectamine 2000 transfection agent (Invitrogen). PMs were washed with sterile 1 × PBS and then cultured in RPMI1640 medium; 2.5 μg of the constructed pcDNA3.1-*vspas7* expression vector was added to 125 μl MEM medium and mixed thoroughly for plasmid dilution. At the same time, 5 μl Lipofectamine 2000 transfection agent was added to 125 μl MEM medium and mixed thoroughly for transfection reagent dilution. Afterwards, the diluted constructed vector was added to the transfection agent and mixed together, left to stand at room temperature (RT) for 5 min and then added to the cells and mixed. After 24 h transfection, PMs were washed with sterile 1 × PBS to remove the liposomal DNA complex and replaced with RPMI1640 medium to continue the culture for subsequent experiments.

### *Giardia intestinalis* stimulated mouse peritoneal macrophages

*Giardia intestinalis* trophozoites (WB strain, ATCC30957; American Type Culture Collection, Manassas, VA, USA) were cultured to logarithmic growth stage in modified TYI-S-33 medium and collected by centrifugation at 1000*g* for 10 min after 30 min in an ice bath. The precipitate was resuspended in RPMI1640 medium and collected by centrifugation at 1000*g* for 10 min again. After the precipitate was suspended in RPMI1640 medium, parasites were counted using a hemocytometer. The PMs were then stimulated with *Giardia* (MOI = 1:1), cell supernatants, and lysates were harvested.

### Western blot

Cell supernatants and precipitates were collected for different times. Supernatants were concentrated using chloroform–methanol. Cellular protein was obtained via the precipitate lysis by RIPA lysis buffer (Beyotime Biotechnology, Shanghai, China) with 1 mM PMSF solution (Beyotime Biotechnology, Shanghai, China), and protein concentration was detected using bicinchoninic acid assay. Equivalent protein levels from the different samples were denatured by mixing with 5 × protein loading buffer (TransGen, Beijing, China) analyzed by sodium dodecyl sulfate–polyacrylamide gel (SDS-PAGE) and then transferred into polyvinyl difluoride membranes (PVDF, Millipore, Billerica, MA, USA). After blocking with 5% skim milk, primary antibodies directed against phospho-p38, phospho-ERK, phospho-p65, phospho-IκBα, p38, ERK, NF-κB p65, GSDMD (all rabbit derived, diluted 1/1000) and IκBα, caspase-1, NLRP3, β-tubulin (all mouse derived, diluted 1/1000) and IL-1β (goat derived, diluted 1/800) were treated with PVDF membranes overnight at 4 °C. Expression of VSPAS7 was detected using His-tag antibody (Proteintech, Wuhan, China). After overnight incubation, PVDF membranes were cleaned and treated with secondary horseradish peroxidase (HRP)-conjugated goat anti-mouse immunoglobulin G (IgG), HRP-conjugated goat anti-rabbit IgG or HRP-conjugated rabbit anti-goat IgG antibodies (Proteintech, Wuhan, China). Membranes were cleaned, and enhanced chemiluminescence reagent (Vigorous, Beijing, China) was employed for detection. Relative grayscale intensity analysis of protein bands was carried out by the ImageJ software (NIH image software).

### Enzyme-linked immunosorbent assays

Cellular supernatants of the different groups were obtained, and then secretions of the pro-inflammatory cytokines interleukin (IL)-6, IL-12 p40, IL-1β, and tumor necrosis factor alpha (TNF-α) were detected with enzyme-linked immunosorbent assays (ELISA) kits (Invitrogen, CA, USA) following the directions provided by the manufacturer. The absorbance was harvested at 450 nm, and standard curves were generated using a microplate reader.

### Lactate dehydrogenase assays

Cellular supernatants were used to detect the detailed lactate dehydrogenase (LDH) procedure obeying the guidelines supplied by the manufacturer (Beyotime Biotechnology, Shanghai, China). The absorbance was measured at 490 nm by a microplate reader.

### Preparation of VSPAS7 primary antibody

The *vspas7* gene was linked to the pET-28a vector to construct the prokaryotic expression vector known as pET-28a-*vspas7*. To obtain VSPAS7 protein, the pET-28a-*vspas7* expression vector was induced and purified. Two-month-old New Zealand female rabbits were immunized two times at 14-day intervals by using an equal mixture of VSPAS7 protein and Freund's adjuvant [31]. After vaccination, rabbit serum was collected, and anti-VSPAS7 antibody was obtained by measuring antibody efficacy.

### Subcellular localization of the *Giardia* VSPAS7 protein

To detect the subcellular localization of VSPAS7, *Giardia* attached to 24-well plates after pretreatment of slides with poly-d-lysine. After 2 h, the *Giardia* were washed and fixed in 4% paraformaldehyde solution. *Giardia* were permeabilized with 0.25% Triton X-100, blocked with 3% BSA and then reacted with primary antibody (diluted 1/200) at 4 °C overnight. After washing, *Giardia* was incubated with Corelite488-conjugated goat anti-rabbit IgG (H + L) (diluted 1/100, Proteintech, Wuhan, China). Hoechst 33342 (Beyotime Biotechnology, Shanghai, China) was used to stain cell nuclei. Under a fluorescence microscope (Olympus, Tokyo, Japan), VSPAS7's subcellular localization was detected.

### Immunofluorescence assays

To detect the subcellular localization of VSPAS7 and NLRP3, PMs attached to 24-well plates incubated with RPMI1640 at 37℃, 5% CO_2_ for 24 h were transfected with the pcDNA3.1-*vspas7* vector and then treated with *Giardia* (MOI = 1:1). After 18 h, the cells were washed and fixed in 4% paraformaldehyde solution. Cells were permeabilized with 0.25% Triton X-100, blocked with 3% BSA and then reacted with primary antibody (diluted 1/200) at 4 °C overnight. After washing, cells were incubated with Corelite488-conjugated goat anti-rabbit IgG (H + L) and Corelite594-conjugated goat anti-mouse IgG (H + L), respectively (diluted 1/100, Proteintech, Wuhan, China). Hoechst 33,342 (Beyotime Biotechnology, Shanghai, China) was used to stain cell nuclei. Under a fluorescence microscope (Olympus, Tokyo, Japan), VSPAS7 and NLRP3's subcellular localization was detected.

### Co-immunoprecipitation

To verify the interaction between VSPAS7 and NLRP3 by co-immunoprecipitation (Co-IP), the overexpression plasmids His-VSPAS7 and Flag-NLRP3 were co-transfected into HEK293T cells. The cells were collected and treated by cell lysis buffer for Western and IP (Beyotime Biotechnology, Shanghai, China) with 1 mM PMSF solution. Then, the cells were centrifuged at 13,000*g* for 10 min, and the supernatants were incubated with anti-His and anti-Flag antibodies or IgG (Proteintech, Wuhan, China) overnight at 4 °C with shaking. The immune complex solution was incubated with protein A/G magnetic beads for 1 h at RT with mixing. The bound immune complex was dissociated from the beads with low-pH buffer and denatured with 1 × SDS buffer. The supernatants were collected and proceeded to western blot assays.

### Bimolecular fluorescence complementation assays

We designed primers to link the bimolecular fluorescent complementary (BiFC) experimental plasmids to targets genes (*Giardia vspas7* gene and mouse *nlrp3* gene) using pBiFC-bFosVC155 and pBiFC-bJunVN173 plasmids as positive controls. The Fos and Jun motifs were replaced with *nlrp3* and *vspas7* genes using seamless cloning as experiment groups, while pBiFC-VC155 and pBiFC-VN173 plasmids were used as negative controls. HEK293T cells were incubated on slides in 24-well cell culture plates and transfected with the above vectors via Lipofectamine 2000 transfection agent. After 24 h incubation, fluorescence was observed via laser confocal fluorescence microscopy.

### Statistical analysis

All results were presented as the mean ± standard error of three biological replicates. Statistics were generated using GraphPad Prism 8.0 software. Multiple groups were compared using one-way ANOVA test. Significance was shown as *p < 0.05, **p < 0.01, ***p < 0.001 and ns indicates “not significant” (*p* > 0.05).

## Results

### VSPAS7 overexpression inhibited the *Giardia*-activated NF-κB and ERK/MAPK pathways, but had no effect on *Giardia*-activated p38 MAPK pathway

The *vspas7* gene was successfully obtained by PCR amplification and ligated to the pcDNA3.1 eukaryotic expression vector. The successful construction of recombinant expression vector was verified via sequencing and BLAST analysis.

**Fig. 1:**
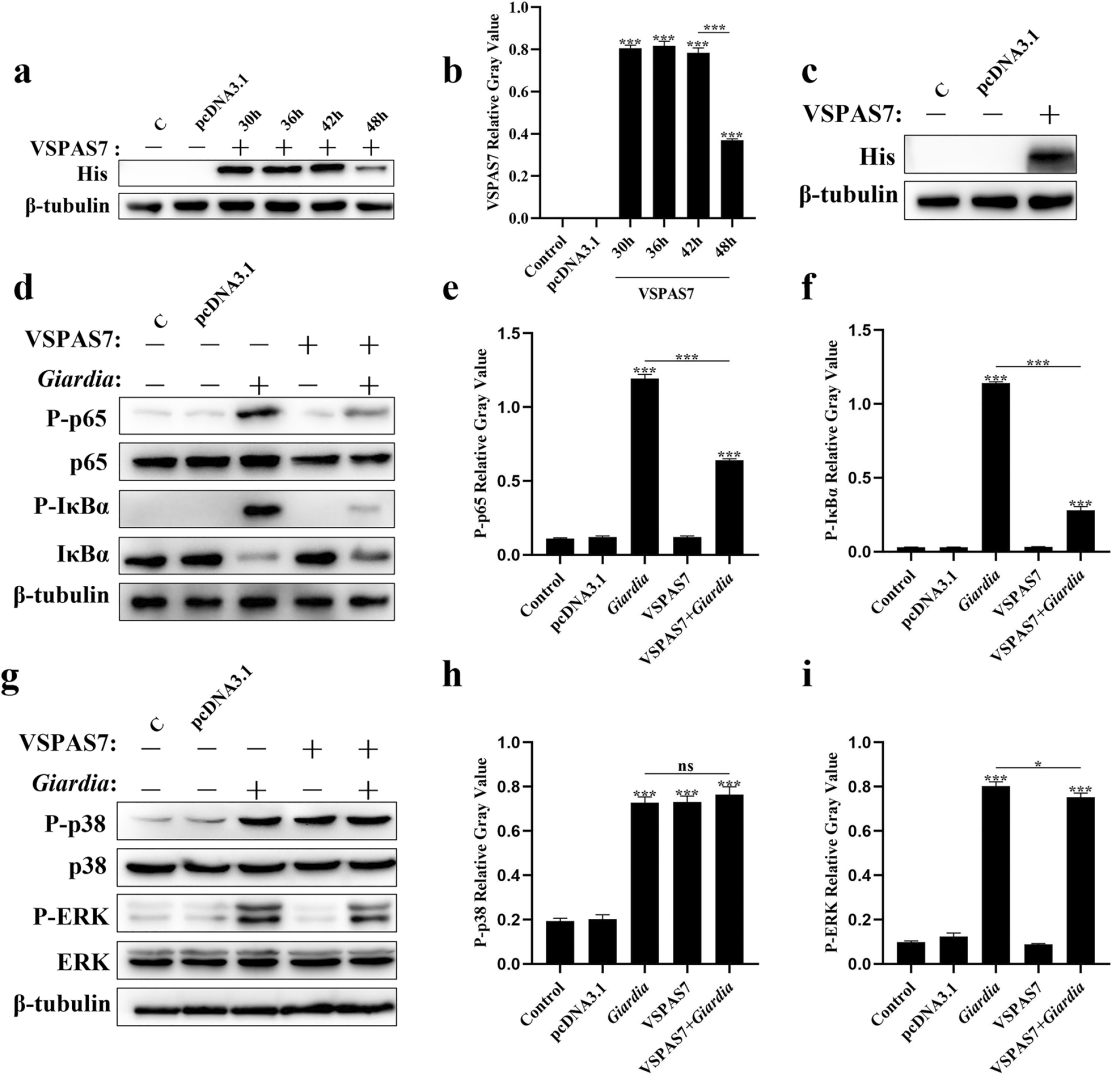
Role of VSPAS7 in *Giardia*-induced NF-κB and MAPK signaling pathways. Western blot analysis (**a**) and relative gray values (**b**) of VSPAS7 expression were performed in mouse PMs transfected with the pcDNA3.1-*vspas7* vector at different times after transfection (30 h, 36 h, 42 h and 48 h). Mouse PMs pre-transfected with pcDNA3.1-*vspas7* vector for 24 h were incubated with *Giardia* trophozoites (MOI = 1:1) for 1 h; then, VSPAS7 expression level (**c**) and NF-κB p65 and IκBα phosphorylation levels were detected by western blot (**d**) and relative gray values (**e, f**). Similarly, mouse PMs pre-transfected with pcDNA3.1-*vspas7* vector for 24 h were incubated with *Giardia* trophozoites (MOI = 1:1) for 3 h, following which p38 and ERK phosphorylation levels were analyzed by western blot (**g**) and relative gray values (**h, i**). C: Control. Results are expressed as mean ± SD from three separate experiments. ns, no significant difference, *p < 0.05, **p < 0.01, ***p < 0.001

The mouse PMs were transfected with pcDNA3.1 and pcDNA3.1-*vspas7* eukaryotic vectors, and cell lysates were harvested at 30 h, 36 h, 42 h and 48 h transfection, separately. Western blot was performed to detect the expression of VSPAS7 (protein expressed by pcDNA3.1-*vspas7* eukaryotic expression vector). The results showed that the *Giardia* VSPAS7 protein could be expressed in mouse macrophages (pcDNA3.1: ANOVA, *F*_(5, 12)_ = 1.300, *p* > 0.9999; 30 h: ANOVA, *F*_(5, 12)_ = 1.300, *p* < 0.0001; 36 h: ANOVA, *F*_(5, 12)_ = 1.300, *p* < 0.0001; 42 h: ANOVA, *F*_(5, 12)_ = 1.300, *p* < 0.0001; 48 h: ANOVA, *F*_(5, 12)_ = 1.300, *p* < 0.0001) and the expression decreased at 42 h of transfection (ANOVA, *F*_(5, 12)_ = 1.300, *p* < 0.0001) (Fig. 1a, b). Interestingly, VSPAS7 protein decreased at 42 h; it remained undegraded by 48 h (see Additional file 1: Fig. S1).

To identify the effect of VSPAS7 on the inflammatory response induced by *Giardia*, we studied whether *Giardia*-induced inflammatory response pathways’ activation would be regulated by VSPAS7. Western blot results showed that the VSPAS7 could be expressed in mouse macrophages (Fig. 1c), and in *Giardia*-stimulated group, the levels of phosphorylated-p65 (P-p65) (ANOVA, *F*_(4, 10)_ = 1.018, *p* < 0.0001) and P-IκBα (ANOVA, *F*_(4, 10)_ = 0.8055, *p* < 0.0001) were increased (Fig. 1d, e and f), suggesting that *Giardia* could lead the NF-κB signaling pathway activation of macrophages. P-p38 upregulation (ANOVA, *F*_(4, 10)_ = 0.3250, *p* < 0.0001) and P-ERK upregulation (ANOVA, *F*_(4, 10)_ = 0.5796, *p* < 0.0001) were also observed in the *Giardia*-stimulated group (Fig. 1g–i), revealing that *Giardia* could activate the ERK/p38 MAPK signaling pathway. Additionally, the VSPAS7 expression group was able to increase p38 phosphorylation levels (ANOVA, *F*_(4, 10)_ = 0.3250, *p* < 0.0001) (Fig. 1g, h) but had no impact on the phosphorylation of ERK (ANOVA, *F*_(4, 10)_ = 0.5796, *p* = 0.9150), p65 (ANOVA, *F*_(4, 10)_ = 1.018, *p* = 0.9544) and IκBα (ANOVA, *F*_(4, 10)_ = 0.8055, *p* > 0.9999) (Fig. 1e, f and i). Compared to the *Giardia*-stimulated group, the levels of P-p65 and P-IκBα were decreased in the VSPAS7 overexpression in the *Giardia*-stimulated group (P-p65: ANOVA, *F*_(4, 10)_ = 1.018, *p* < 0.0001; P-IκBα: ANOVA, *F*_(4, 10)_ = 0.8055, *p* < 0.0001) (Fig. 1d–f), revealing that VSPAS7 overexpression could inhibit activation of NF-κB signaling pathway caused by *Giardia*. We also found that in the MAPK pathway, compared to the *Giardia*-stimulated group, the level of P-ERK was decreased in VSPAS7 overexpression in the *Giardia*-stimulated group (ANOVA, *F*_(4, 10)_ = 0.5796, *p* = 0.0149) (Fig. 1g, i), which indicated that VSPAS7 overexpression could inhibit the activation of ERK/MAPK signaling pathway induced by *Giardia*. However, VSPAS7 overexpression had no impact on p38 phosphorylation caused by *Giardia* (ANOVA, *F*_(4, 10)_ = 0.3250, *p* = 0.4320) (Fig. 1g, h).

**Fig. 2:**
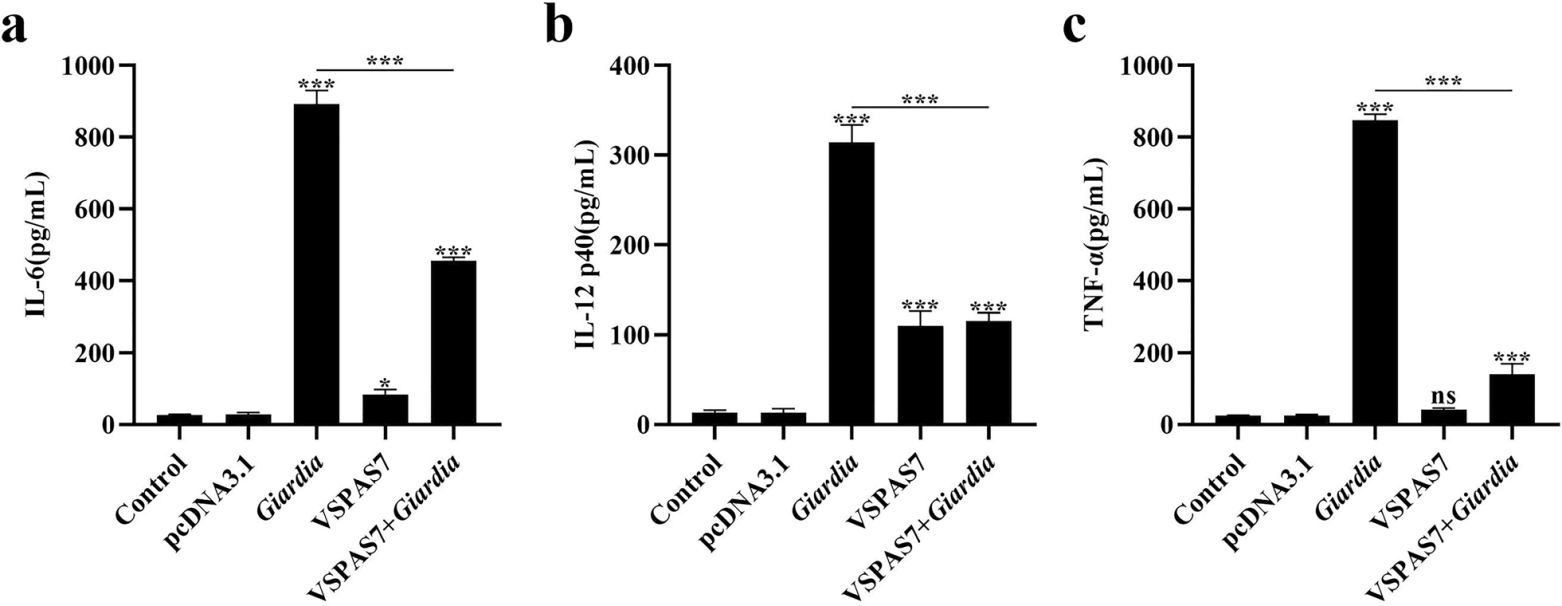
Role of VSPAS7 in *Giardia*-stimulated pro-inflammatory cytokine release from mouse PMs. After transfection with the pcDNA3.1-*vspas7* vector for 24 h, mouse PMs were stimulated with *Giardia* trophozoites (MOI = 1:1), and the culture supernatant was collected after 18 h. The secretion levels of IL-6 (**a**), IL-12 (**b**) and TNF-α (**c**) in the supernatant were measured by ELISA. Results are expressed as mean ± SD from three separate experiments. ns, no significant difference, *p < 0.05, **p < 0.01, ***p < 0.001

ELISA results showed that the *Giardia* induced a highly significant increase in the secretion of IL-6, IL-12 p40 and TNF-α compared to the control group (IL-6: ANOVA, *F*_(4, 10)_ = 1.116, *p* < 0.0001; IL-12 p40: ANOVA, *F*_(4, 10)_ = 0.6885, *p* < 0.0001; TNF-α: ANOVA, *F*_(4, 10)_ = 1.990, *p* < 0.0001) (Fig. 2a–c). VSPAS7 could cause the release of IL-6 and IL-12 p40 (IL-6: ANOVA, *F*_(4, 10)_ = 1.116, *p* = 0.0294; IL-12 p40: ANOVA, *F*_(4, 10)_ = 0.6885, *p* < 0.0001) but not the secretion of TNF-α (Fig. 2a–c). Also, VSPAS7 overexpression in the *Giardia*-stimulated group significantly decreased the secretion levels of IL-6, IL-12 p40 and TNF-α in PMs compared to the *Giardia*-stimulated group (IL-6: ANOVA, *F*_(4, 10)_ = 1.116, *p* < 0.0001; IL-12 p40: ANOVA, *F*_(4, 10)_ = 0.6885, *p* < 0.0001; TNF-α: ANOVA, *F*_(4, 10)_ = 1.990, *p* < 0.0001) (Fig. 2a–c), indicating that VSPAS7 could also cause changes in downstream cytokine levels.

These data indicated that VSPAS7 overexpression attenuated *Giardia*-activated immune responses of host macrophage by inhibiting NF-κB and ERK/MAPK signaling pathways, which resulted in the reduction of IL-6, IL-12 p40 and TNF-α pro-inflammatory cytokine production levels brought on by *Giardia*.

### VSPAS7 overexpression inhibited *Giardia*-induced host macrophage pyroptosis

There was evidence that NF-κB and MAPK pathways could induce further inflammasome activation [32]. To elucidate whether VSPAS7 overexpression inhibited *Giardia*-induced inflammatory responses in host cells by further regulating the inflammasome activation and thus affecting macrophage pyroptosis, the expression or activation levels of key proteins, the secretion levels of IL-1β and the LDH release were examined.

**Fig. 3:**
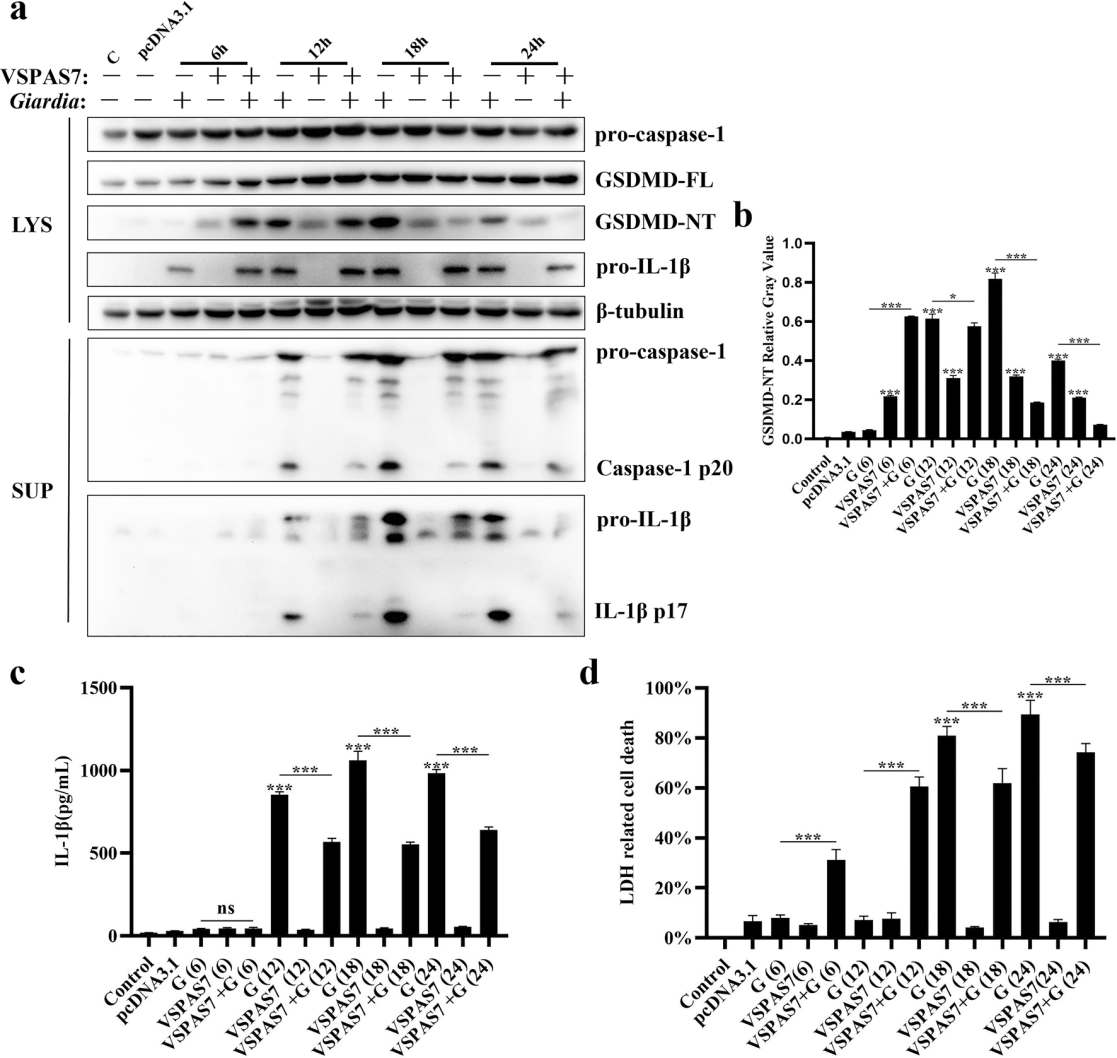
Role of VSPAS7 in *Giardia*-induced host macrophage pyroptosis. Mouse PMs pre-transfected with pcDNA3.1-*vspas7* vector for 24 h were incubated with *Giardia* trophozoites (MOI = 1:1). Cell culture supernatants and precipitates were harvested at different *Giardia* stimulation times (6 h, 12 h, 18 h and 24 h). Cells transfected with the pcDNA3.1 vector were regarded as the negative control and the *Giardia*-stimulated group as the positive control. **a** Expression or activation levels of key proteins during pyroptosis were measured by western blot. **b** Relative gray values of GSDMD-NT are shown. **c** IL-1β levels in cell supernatants were measured by ELISA. **d** LDH release in cell culture supernatants was measured by LDH assay. C: Control; G: *Giardia*. Results are expressed as mean ± SD from three separate experiments. ns, no significant difference, *p < 0.05, **p < 0.01, ***p < 0.001

The results showed that in *Giardia*-stimulated mouse PMs, the cleavage of GSDMD N-terminal was detected, compared to the control group, and this phenomenon was most evident at 18 h of *Giardia* stimulation (ANOVA, *F*_(13, 28)_ = 1.273, *p* < 0.0001) (Fig. 3a, b). In addition, after 12 h of *Giardia* stimulation, the IL-1β p17 maturation fragment and cleaved caspase-1 p20 fragment were detected in supernatant samples (Fig. 3a) and were greatly increased at 18 h. ELISA results also revealed that secretion of IL-1β greatly increased *Giardia* stimulation at 12 h, 18 h and 24 h (ANOVA, *F*_(13, 28)_ = 0.7178, *p* < 0.0001) (Fig. 3c). Similarly, after 18 h and 24 h of *Giardia* stimulation, increased LDH release was detected in supernatant samples (ANOVA, *F*_(13, 24)_ = 0.6427, *p* < 0.0001) (Fig. 3d) and gradually increased with increasing duration of *Giardia* stimulation. These results suggested that *Giardia* could activate GSDMD-mediated macrophage pyroptosis in mice. IL-1β secretion (Fig. 3c) and LDH release level (Fig. 3d) in the VSPAS7 expression group were not significantly different from those in the control group, so only VSPAS7 was unable to cause PMs pyroptosis.

However, compared to the *Giardia*-stimulated group, the N-terminal cleavage of GSDMD was reduced in the VSPAS7 overexpression with the *Giardia*-stimulated group at 12 h (ANOVA, *F*_(13, 28)_ = 1.273, *p* = 0.0384), and the difference was most pronounced at 18 h and 24 h (ANOVA, *F*_(13, 28)_ = 1.273, *p* < 0.0001) (Fig. 3a, b). Meanwhile, the IL-1β p17 maturation fragment and the cleaved caspase-1 p20 were reduced in supernatant samples from 12 h (Fig. 3a). The results of ELISA and LDH assays showed that in the VSPAS7 overexpression with the *Giardia*-stimulated group IL-1β secretion was reduced from 12 h (ANOVA, *F*_(13, 28)_ = 0.7178, *p* < 0.0001) (Fig. 3c) and the LDH release was reduced from 18 h (ANOVA, *F*_(13, 24)_ = 0.6427, *p* < 0.0001) (Fig. 3d) compared to that in the *Giardia*-stimulated group. Although VSPAS7 itself could not induce pyroptosis in mouse PMs, it could regulate *Giardia*-induced pyroptosis. To sum up, overexpression of VSPAS7 could attenuate *Giardia*-induced pyroptosis by inhibiting the cleavage of GSDMD activated by *Giardia*.

### Overexpression of VSPAS7 inhibited *Giardia*-induced host macrophage pyroptosis via NLRP3/caspase-1/GSDMD

*Giardia* could trigger mouse PM pyroptosis through NLRP3 inflammasome activation [13]. In our study, it was discovered that VSPAS7 overexpression could inhibit *Giardia*-induced GSDMD cleavage and caspase-1 activation. Moreover, NLRP3 functions as an upstream molecule in the GSDMD and caspase-1 classical pathway. Therefore, this section examined whether NLRP3 inflammasome is involved in the suppression of *Giardia*-induced pyroptosis by VSPAS7 using NLRP3-deficient cells. By measuring the expression or activation of key proteins, release of IL-1β and level of LDH, it was possible to assess the impact of NLRP3 on VSPAS7-regulated *Giardia*-activated pyroptosis.

**Fig. 4:**
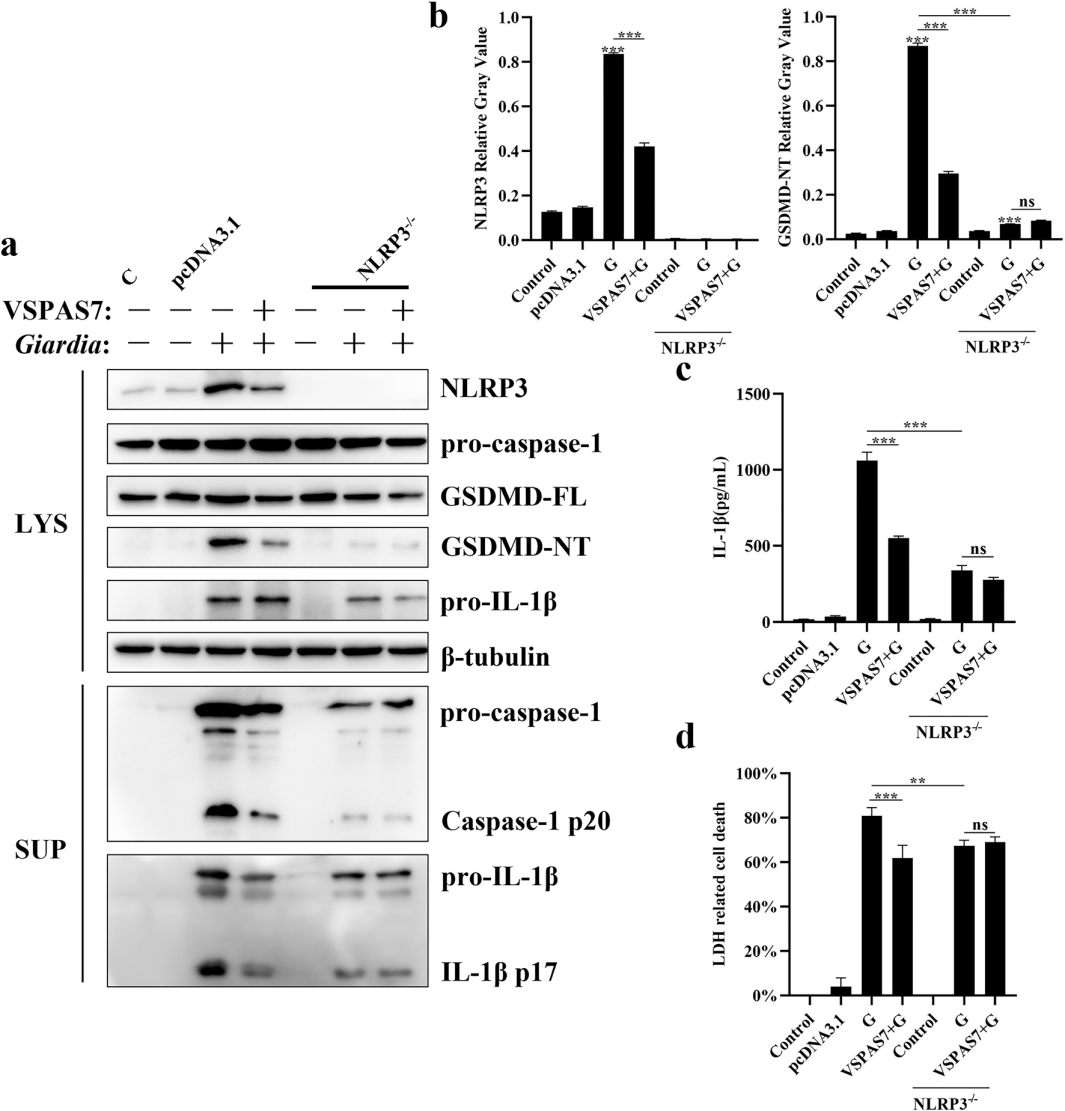
Role of NLRP3 in the inhibition of *Giardia*-induced host macrophage pyroptosis by VSPAS7. Both WT and NLRP3-deficient (*NLRP3*^*−/−*^) mouse PMs pre-transfected with pcDNA3.1-*vspas7* vector for 24 h were incubated with *Giardia* trophozoites (MOI = 1:1). Cell supernatants and precipitates were harvested at 18 h. **a** Expression or activation levels of key proteins during pyroptosis were measured by western blot. **b** Relative gray values of NLRP3 and GSDMD-NT are shown. **c** IL-1β levels in cell supernatants were measured by ELISA. **d** LDH release in cell culture supernatants was measured by LDH assay. C: Control; G: *Giardia*. Results are expressed as mean ± SD from three separate experiments. ns, no significant difference, *p < 0.05, **p < 0.01, ***p < 0.001

The results showed that, compared to that in wild-type (WT) cells, *Giardia*-induced GSDMD N-terminal cleavage was reduced (ANOVA, *F*_(6, 14)_ = 1.425, *p* < 0.0001) (Fig. 4a, b), and the levels of IL-1β p17 and cleaved caspase-1 p20 were reduced in supernatant samples (Fig. 4a) in NLRP3-deficient cells. In addition, *Giardia* induced higher levels of IL-1β secretion (ANOVA, *F*_(6, 14)_ = 0.8642, *p* < 0.0001) and LDH release (ANOVA, *F*_(6, 14)_ = 1.309, *p* = 0.0026) in WT cells than in NLRP3-deficient cells (Fig. 4c, d). These results suggested that *Giardia* could trigger NLRP3 inflammasome responses and thus induce pyroptosis in macrophages.

In NLRP3-deficient cells, there were no significant differences in the N-terminal cleavage of GSDMD (ANOVA, *F*_(6, 14)_ = 1.425, *p* = 0.1072) (Fig. 4a, b), IL-1β p17 and cleaved caspase-1 p20 (Fig. 4a) between the VSPAS7 overexpression with the *Giardia*-stimulated group and the *Giardia*-stimulated group. It was different that VSPAS7 overexpression reduced *Giardia*-induced IL-1β secretion in WT PMs (ANOVA, *F*_(6, 14)_ = 0.8642, *p* < 0.0001), while there was no significant difference (ANOVA, *F*_(6, 14)_ = 0.8642, *p* = 0.1318) in the secretion levels of IL-1β between the *Giardia*-stimulated group and VSPAS7 overexpression and the *Giardia*-stimulated group in NLRP3-deficient cells (Fig. 4c). Similarly, overexpression of VSPAS7 inhibited *Giardia*-induced LDH release in WT PMs (ANOVA, *F*_(6, 14)_ = 1.309, *p* < 0.0001), and there was also no significant difference (ANOVA, *F*_(6, 14)_ = 1.309, *p* = 0.9937) in LDH release between the *Giardia*-stimulated group and the VSPAS7 overexpression with the *Giardia*-stimulated group in NLRP3-deficient cells (Fig. 4d). Above all, VSPAS7 overexpression could inhibit *Giardia*-induced macrophage pyroptosis via NLRP3 inflammasome.

### *Giardia* VSPAS7 interacted with mouse NLRP3

**Fig. 5:**
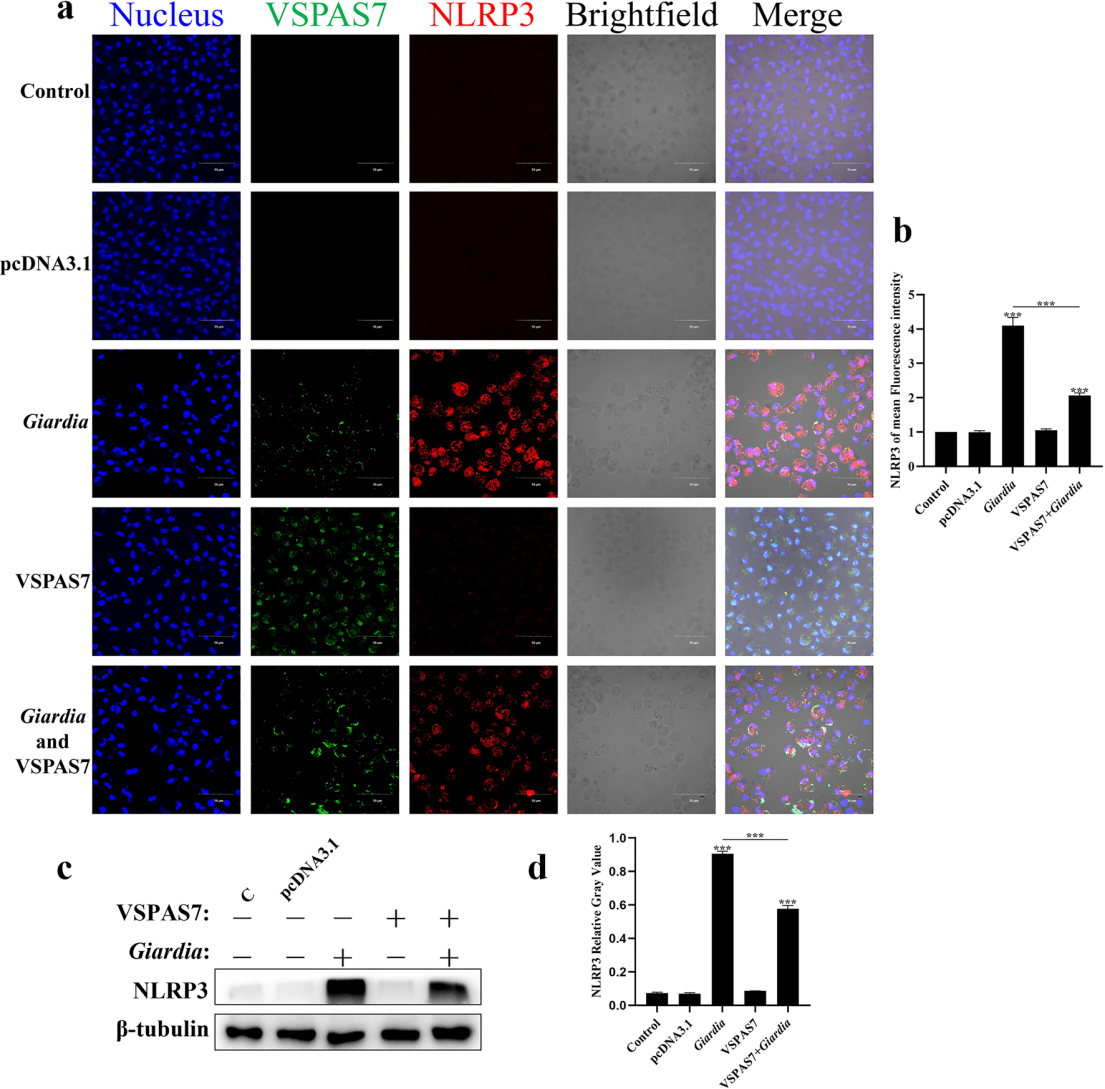
The localization of NLRP3 and VSPAS7. Cell nucleus, NLRP3 and VSPAS7 were detected by laser confocal microscopy in **a**. **b** The mean fluorescence intensity of NLRP3. Blue: Hoechst 33,342. Green: *Giardia* VSPAS7. Red: Mouse NLRP3. Scale bar: 50 μm. **c** Expression level of NLRP3 was measured by Western blot. **d** Relative gray values of NLRP3 are shown. C: Control. Results are expressed as mean ± SD from three separate experiments. ns, no significant difference, *p < 0.05, **p < 0.01, ***p < 0.001

To elucidate the relationship between VSPAS7 and NLRP3 in inhibiting *Giardia*-induced pyroptosis, immunofluorescence assays, co-immunoprecipitation and bimolecular fluorescence complementation assays were used. We first determined the subcellular localization of the VSPAS7 protein on the surface of *Giardia* trophozoites. Immunofluorescence results showed that the protein was located on the surface of the trophozoites (Additional file 1: Fig. S2). Compared to the control group, expression of NLRP3 (red fluorescence) was increased and aggregated in the *Giardia*-stimulated group, indicating that *Giardia* was able to induce NLRP3 aggregation in mouse macrophages (ANOVA, *F*_(4, 10)_ = 2.360, *p* < 0.0001) (Fig. 5a, b). VSPAS7 itself did not induce cellular NLRP3 aggregation (Fig. 5a, b). However, a high level of VSPAS7 expression (green fluorescence) was found in the VSPAS7 overexpression with the *Giardia*-stimulated group, while NLRP3 expression was significantly reduced compared to the *Giardia*-stimulated group (ANOVA, *F*_(4, 10)_ = 2.360, *p* < 0.0001) (Fig. 5a, b); this was in accordance with the result in Fig. 5c, d that NLRP3 expression caused by *Giardia* was inhibited by VSPAS7 overexpression (ANOVA, *F*_(4, 10)_ = 0.9812, *p* < 0.0001). Thus, aggregation of NLRP3 was also attenuated. Interestingly, in the *Giardia*-stimulated group and the VSPAS7 overexpression with the *Giardia*-stimulated group, yellow fluorescence could be observed (Fig. 5a). These results suggested that *Giardia* VSPAS7 potentially interacted with mouse NLRP3 protein and inhibited the expression of NLRP3, thereby inhibiting *Giardia*-induced macrophage pyroptosis through NLRP3 inflammasome.

**Fig. 6:**
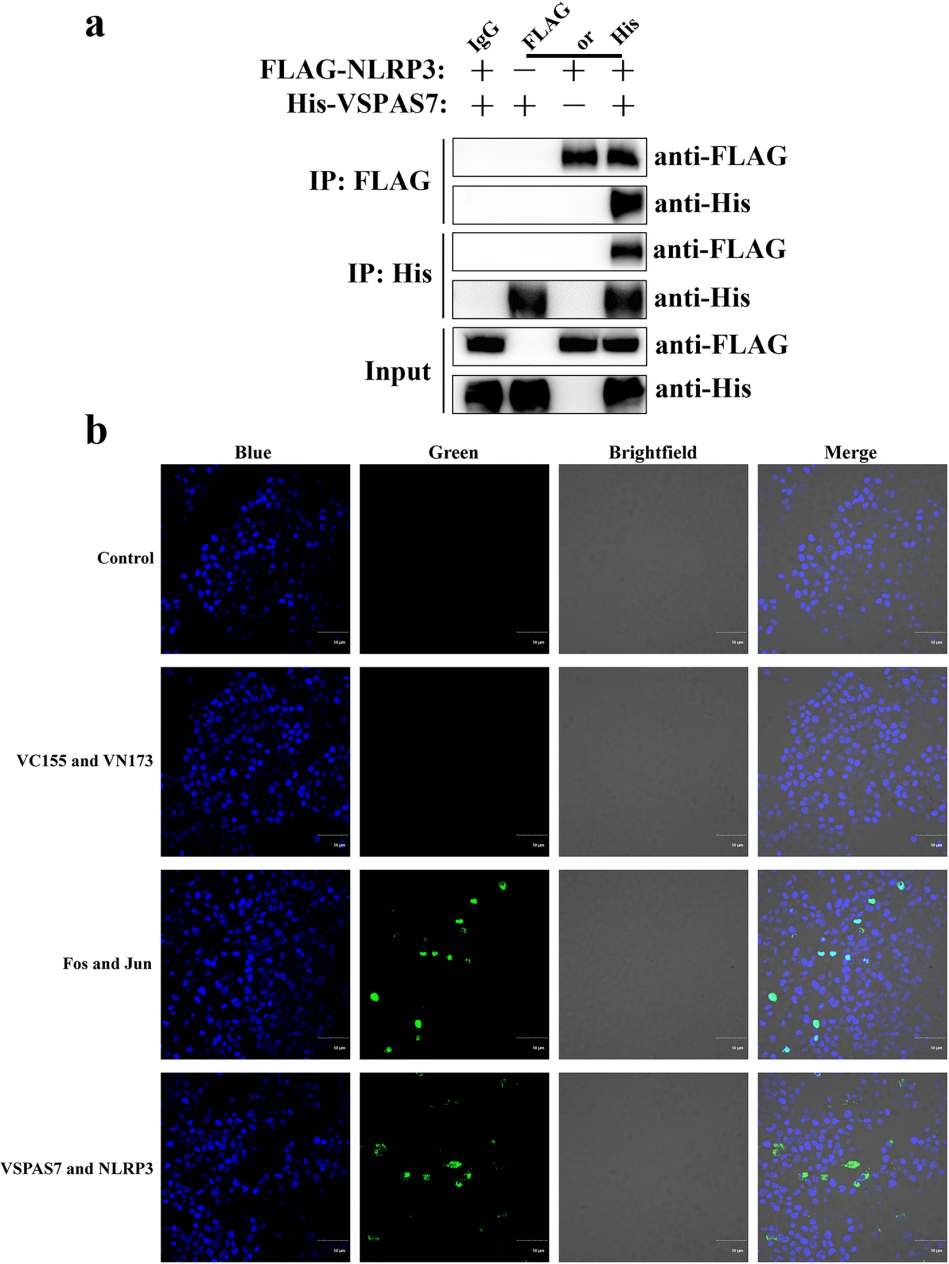
Interaction of mouse NLRP3 and *Giardia* VSPAS7. **a** Co-immunoprecipitation assays of NLRP3 and VSPAS7. HEK293T cells were transfected with pcDNA3.1-N-Flag-*nlrp3* and pcDNA3.1-His-*vspas7* for 24 h. The cellular proteins, anti-His/Flag tag antibody and protein A/G magnetic beads were co-incubated and analyzed by western blot. IgG: A negative control; the cellular proteins co-transfected with pcDNA3.1-N-Flag-*nlrp3* and pcDNA3.1-His-*vspas7*, anti-IgG antibody and protein A/G magnetic beads were co-incubated. **b** Bimolecular fluorescence complementation experiments of mouse NLRP3 and *Giardia* VSPAS7. HEK293T cells were co-transfected with constructed pBiFC-nlrp3VC155 and pBiFC-vspas7VN173 plasmids for 24 h and observed by laser confocal fluorescence microscopy. The co-transfection of pBiFC-bFosVC155 and pBiFC-bJunVN173 plasmids was used as the positive control, while the co-transfection of pBiFC-VC155 and pBiFC-VN173 plasmids was used as the negative control. Blue: Hoechst 33342 staining of HEK293T cell nuclei. Green: green fluorescence emitted upon protein interactions. Scale bar: 50 μm

To determine whether *Giardia* VSPAS7 is correlated with NLRP3, we constructed expression vectors to verify this connection by Co-IP and BiFC assays. In Co-IP assay, proteins were extracted from the cell lysates of each group and analyzed by western blot. The results revealed that the expression of His-tagged protein was detected in the pcDNA3.1-His-*vspas7* transfection group (Fig. 6a), and the expression of Flag-tagged protein was detected in the pcDNA3.1-N-Flag-*nlrp3* transfection group (Fig. 6a), and the pcDNA3.1-His-*vspas7* and pcDNA3.1-N-Flag-*nlrp3* co-transfection group could detect the expression of both His and Flag tag proteins (Fig. 6a), indicating that pcDNA3.1-His-*vspas7* and pcDNA3.1-N-Flag-*nlrp3* could be stably expressed in HEK293T cells. The cellular proteins, anti-His tag antibody and protein A/G magnetic beads were co-incubated and analyzed by Western blot; the results showed that the expression of His tag protein was detected in the pcDNA3.1-His-*vspas7* transfection group, the pcDNA3.1-N-Flag-*nlrp3* transfection group did not detect protein expression, and pcDNA3.1-His-*vspas7* and pcDNA3.1-N-Flag-*nlrp3* co-transfection group could detect expression of both His and Flag tag proteins (Fig. 6a), indicating that VSPAS7 interacted with NLRP3 in eukaryotic cells. Similarly, co-incubation of cellular proteins, anti-Flag tag antibody and protein A/G magnetic beads with Western blot analysis also demonstrated that VSPAS7 interacted with NLRP3 protein (Fig. 6a). In BiFC assays, the *Giardia vspas7* gene and mouse *nlrp3* gene were amplified and successfully ligated to BiFC vectors, respectively. Cells in the control group and co-transfection of pBiFC-VC155 and pBiFC-VN173 plasmid group (negative control) did not express green fluorescence, indicating that protein interaction did not happen (Fig. 6b). In the positive control group, the green fluorescence emitted after co-transfection of pBiFC-bFosVC155 (Fos) and pBiFC-bJunVN173 (Jun) showed protein interaction (Fig. 6b). After HEK293T cells were co-transfected with pBiFC-nlrp3VC155 (NLRP3) and pBiFC-vspas7VN173 (VSPAS7) for 24 h, the green fluorescence intensity was observed by laser confocal fluorescence microscopy (Fig. 6b). These results proved a direct interaction between *Giardia* VSPAS7 and NLRP3 in mouse macrophages.

## Discussion

Macrophages, as the main effector cells of the host’s innate immune system, are involved in the host’s anti-microbial defense system, which is the first line of defense against pathogenic invasion [33]. Based on previous studies [11, 29, 30], we chose mouse peritoneal macrophages as the target cell for the study of host pyroptosis and immune evasion.

Surface antigen variation is one of the main strategies of pathogenic microorganisms to evade the host immune responses, thus maintaining chronic and recurrent infections [34, 35]. In *Giardia*, antigenic variation involves changes in variant-specific surface proteins [36]. Most current studies have found that *Giardia* VSPs can induce host immune responses. For example, VSPs can activate the host innate immune responses in a TLR-4-dependent manner, as well as activate TLR2 [10], and these can be used as protection strategies or adjuvants for oral vaccine antigens [10]. In addition, researchers have demonstrated that VSPs inhibit proteases, resist proteolysis and extremes of pH and temperature, and activate the host humoral immune responses; thus, they are recognized by the serum of *Giardia*-infected individuals [37]. The presence of VSPs can also be detected in ESPs of *Giardia*, which play a crucial role in inducing cytotoxic damage and alteration of small intestinal epithelial cells [38, 39]. Current research on the mechanisms by which VSPs cause *Giardia* to evade the host immune responses have focused on the reliance on VSPs themselves being constantly altered so that they are not recognized by the host. The role of VSPs in *Giardia*-induced host innate immune responses has not been reported yet.

As an extracellular parasite, *Giardia* trophozoites are mainly colonized in the duodenum, and host cell regulation by trophozoites is partly accomplished through secreted EVs. EVs released by *Giardia* can regulate the host’s immune responses via TLR2 and NLRP3 inflammasome [11], while NF-κB and MAPK signaling pathways are often involved in immune regulation as intermediate pathways between TLR2 downstream and NLRP3 upstream [40, 41]. According to our previous study, the EVs released by *Giardia* contain a variety of proteins, and proteomic analysis of the EVs revealed that they contain VSP proteins [11]. This led us to suspect that VSP proteins may enter host cells via the extracellular vesicle pathway and participate in the regulation of host innate immune responses. Therefore, we first verified whether VSP exerted regulatory effects on the NF-κB and MAPK signaling pathways of TLR2 downstream in host cells and found that overexpression of VSPAS7 could inhibit the activation of host NF-κB and ERK/MAPK signaling pathways by *Giardia*. During *Giardia* infection, the expression of VSPAS7 protein in EVs can effectively inhibit the activation of NLRP3 inflammasomes and NF-κB signaling pathway in macrophages, inhibiting pyroptosis. Therefore, the expression of VSPAS7 protein facilitates the escape from macrophage immunocide during *Giardia* infection, permitting *Giardia* to multiply within the host and increase the parasite burden at the infection site. In a previous study, *Shigella* ubiquitin ligase IpaH7.8 targeted Gasdermin D for degradation to prevent pyroptosis and enhance the bacterial replication in the infected organ [42]. Therefore, we hypothesize that the VSPAS7 protein may also inhibit macrophage death as a result of *Giardia*-host co-evolution and help *Giardia* to reproduce in the host, but the specifics need to be further explored in future experiments. We discovered in our research that VSPAS7 alone could phosphorylate p38. The host's innate immune system could be stimulated by VSP1267, VSP9B10 and VSPH7 in a TLR4-dependent manner [10]. It has been demonstrated that pathogens can activate p38 MAPK as a downstream pathway of TLR4 [43]. This could be the mechanism via which VSPAS7 phosphorylated p38. That VSPAS7 alone could enhance p38 phosphorylation, while overexpression of VSPAS7 had no impact on phosphorylation of p38 by *Giardia*, also needs to be studied. According to our study, *Giardia* induced a significant increase in the secretion of IL-6, IL-12 p40 and TNF-α, and VSPAS7 itself could cause the release of IL-6 and IL-12 p40. However, VSPAS7 overexpression significantly decreased the secretion levels of IL-6, IL-12 p40 and TNF-α caused by *Giardia*. This might be because when only VSPAS7 worked on the cells, it was able to activate p38 phosphorylation. In contrast, when VSPAS7 was overexpressed with the *Giardia*-stimulated cells, VSPAS7 overexpression was able to inhibit the phosphorylation of p65, IκBα and ERK activated by *Giardia*. This suggested that VSPAS7 could affect a wider range of pathways when working together with *Giardia* and therefore produced different results than VSPAS7 alone.

As the first initiation signal of the pyroptosis classical pathway, NF-κB signaling pathway will complete the assembly of classical inflammasomes [44]. *Giardia* is known to induce host macrophage pyroptosis [13]; we wanted to determine whether VSPAS7 was involved in mediating the classical pathway of *Giardia*-induced pyroptosis in host macrophage while regulating the NF-κB signaling pathway. Our study found that overexpression of VSPAS7 could significantly reduce *Giardia*-induced cleavage of GSDMD, which is the pyroptosis marker. The secretion of IL-1β and the release of LDH at 18 h and 24 h demonstrated that VSPAS7 overexpression could inhibit *Giardia*-induced host PMs pyroptosis. According to our findings, *Giardia* was unable to induce GSDMD N-terminal cleavage, IL-1β secretion and LDH release at 6 h of *Giardia* stimulation of peritoneal macrophages. This implied that host macrophage pyroptosis could not be induced at this point. In contrast, when VSPAS7 was overexpressed with the *Giardia*-stimulated cells at this time, VSPAS7 overexpression was able to enhance GSDMD N-terminal cleavage of macrophages by *Giardia*, and VSPAS7 itself could also induce GSDMD N-terminal cleavage, therefore possibly because of the protein's own ability to cause cellular GSDMD N-terminal cleavage at 6 h. At 6 h and 12 h of *Giardia* stimulation, VSPAS7 overexpression was able to promote the release of LDH from *Giardia*-stimulated cells, possibly by regulating other modes of cell death and leading to membrane damage.

As signaling transduction protein complexes, inflammasomes are capable of receiving both endogenous and exogenous stimuli. Among them, NLRP3 inflammasomes are the most widely studied [45]. NLRP3/caspase-1/GSDMD form the classical pathway of pyroptosis and are popular for studying this death mode [46, 47]. According to previous studies and results we obtained, VSPAS7 overexpression could inhibit the activation of the host macrophage NF-κB signaling and pyroptosis caused by *Giardia*, but whether the cause of this phenomenon was related to NLRP3 was not known. Subsequently, the NLRP3-deficient PM experiment showed that overexpression of VSPAS7 was unable to play a role in *Giardia*-induced cleavage of GSDMD, secretion of IL-1β and LDH release. This indicates that VSPAS7 acts through NLRP3 inflammasome to inhibit *Giardia*-induced pyroptosis.

There are three pathways of pyroptosis: the classical, non-classical and novel pathways. Of these, the classical pathway is most widely studied. Common inflammasomes in the classical pathway include NLRP3, NLRC4 and AIM2 inflammasome [18]. This article focuses on the effect of *Giardia* and its proteins on the classical pathway of pyroptosis (NLRP3/caspase-1/GSDMD). Currently, it has been demonstrated that *Giardia* can induce host macrophage pyroptosis through activation of NLRP3 inflammasome, while it is unclear whether it can work via other inflammasomes and pathways, which may also be the reason why *Giardia* can lead to cellular LDH release in NLRP3-deficient cells.

It has been shown that NEK7 (mitotic kinase) interacts with NLRP3 to regulate pyroptosis in inflammatory bowel disease (IBD) through the NF-κB signaling pathway [44]. For VSPAS7, as a regulator of *Giardia*-induced pyroptosis via NLRP3 inflammasome, yellow fluorescence was observed in immunofluorescence assays, which indicated that NLRP3 and VSPAS7 were co-localized. In addition, after PMs were stimulated with *Giardia*, VSPAS7 could be found in the cytoplasm of PMs, implying that VSPAS7 could be secreted from *Giardia* and enter host cells, but whether this process relies on EVs or other detailed mechanisms needs to be additionally explored in the future. The immunofluorescence assays and the observation that *Giardia*-induced pyroptosis was not inhibited by VSPAS7 in NLRP3-deficient cells indicated that VSPAS7 interacted with NLRP3. Next, Co-IP and BiFC assays suggested that VSPAS7 and NLRP3 had a direct interaction in eukaryotic cells. These indicated that NLRP3 was the key protein involved for VSPAS7 to inhibit *Giardia*-induced host macrophage pyroptosis. For the detailed interactions between NLRP3 and VSPAS7, more research is still required. NLRP3 is currently linked to the occurrence of numerous illnesses [48]. Whether VSPAS7 interacts with NLRP3 to impact other host reactions is still not known.

The attenuating effect of VSPAS7 on *Giardia*-activated host macrophage pyroptosis was an important player and enforcer of *Giardia*-regulated host innate immune responses, not only by modulating the NF-κB pathway to inhibit pyroptosis, but also by acting directly on NLRP3 inflammasome. The first discovery that *Giardia* protein inhibits *Giardia*-induced host pyroptosis may provide further insight into the complex mechanisms of pathogenic regulation of host cells, providing new targets and research directions for drug development in bovine and sheep giardiasis [49]. However, this complexity is still unknown. We have only studied VSPAS7. Whether other VSPs of *Giardia* have regulatory effects on pyroptosis remains to be identified, which requires further study.

Furthermore, the distribution of VSPAS7 expressed from pcDNA3.1-*vspas7* exhibited slight variations compared to that of EVs in the cytoplasm of macrophages. To enhance the stimulation model in our subsequent experiments, we can consider refining the approach. For instance, we can extract the VSPAS7 protein from *Giardia*-derived EVs or develop a system where VSPAS7 is exclusively encapsulated within EVs and then used to infect macrophages. These modifications will enable us to delve deeper into understanding the precise role and interactions of VSPAS7 with macrophages. As we proceed with the further assay, we recognize the need for extensive exploration to uncover new insights.

## Conclusion

In summary, we demonstrated that overexpression of VSPAS7 could inhibit *Giardia*-induced pyroptosis. The reason for this effect was, on the one hand, the ability of VSPAS7 to attenuate *Giardia*-activated host NF-κB signaling pathway and, on the other, to interact with the host NLRP3 protein. Both were able to influence the activity of host macrophage pyroptosis via the NLRP3/caspase-1/GSDMD classical inflammasome pathway. Our study demonstrated that *Giardia* VSPAS7 protein could indirectly induce immune evasion by attenuating *Giardia*-induced host macrophage pyroptosis for the first time, providing a new research direction and target for the prevention and treatment of sheep and bovine giardiasis.

### Supplementary Information

**Additional file 1: Fig. S1.** Expression level of VSPAS7. **Fig. S2.** Localization of VSPAS7 in *Giardia*.

## Data Availability

The data that support the findings of this study are available from the corresponding author, Xiaocen Wang: wangxiaocen2016@163.com; Pengtao Gong: gongpt@jlu.edu.cn.

## Abbreviations

VSPs: Variant-specific surface proteins
NLRP3: Nucleotide-binding oligomerization-like receptor 3
ASC: Apoptosis speck-like protein
EVs: Extracellular vesicles
IL-1β: Interleukin-1 beta
PAMPs/DAMPs: Pathogen/damage-associated molecular patterns
PRR: Pattern recognition receptor
NF-κB: Nuclear factor kappa-B
MAPK: Mitogen-activated protein kinase
ERK: Extracellular regulated protein kinase
