# Supplementary material for: Giardia VSPAS7 protein attenuates Giardia intestinalis-induced host macrophage pyroptosis
Source: Parasit Vectors. 2023 Oct 11;16:359. doi: 10.1186/s13071-023-05949-0 (PMC10566177; doi:10.1186/s13071-023-05949-0)
Supplement: Supplementary file 1 — Additional file 1: Fig. S1. Expression level of VSPAS7. Fig. S2. Localization of VSPAS7 in Giardia. [file 13071_2023_5949_MOESM1_ESM.docx]

**Additional Material：**


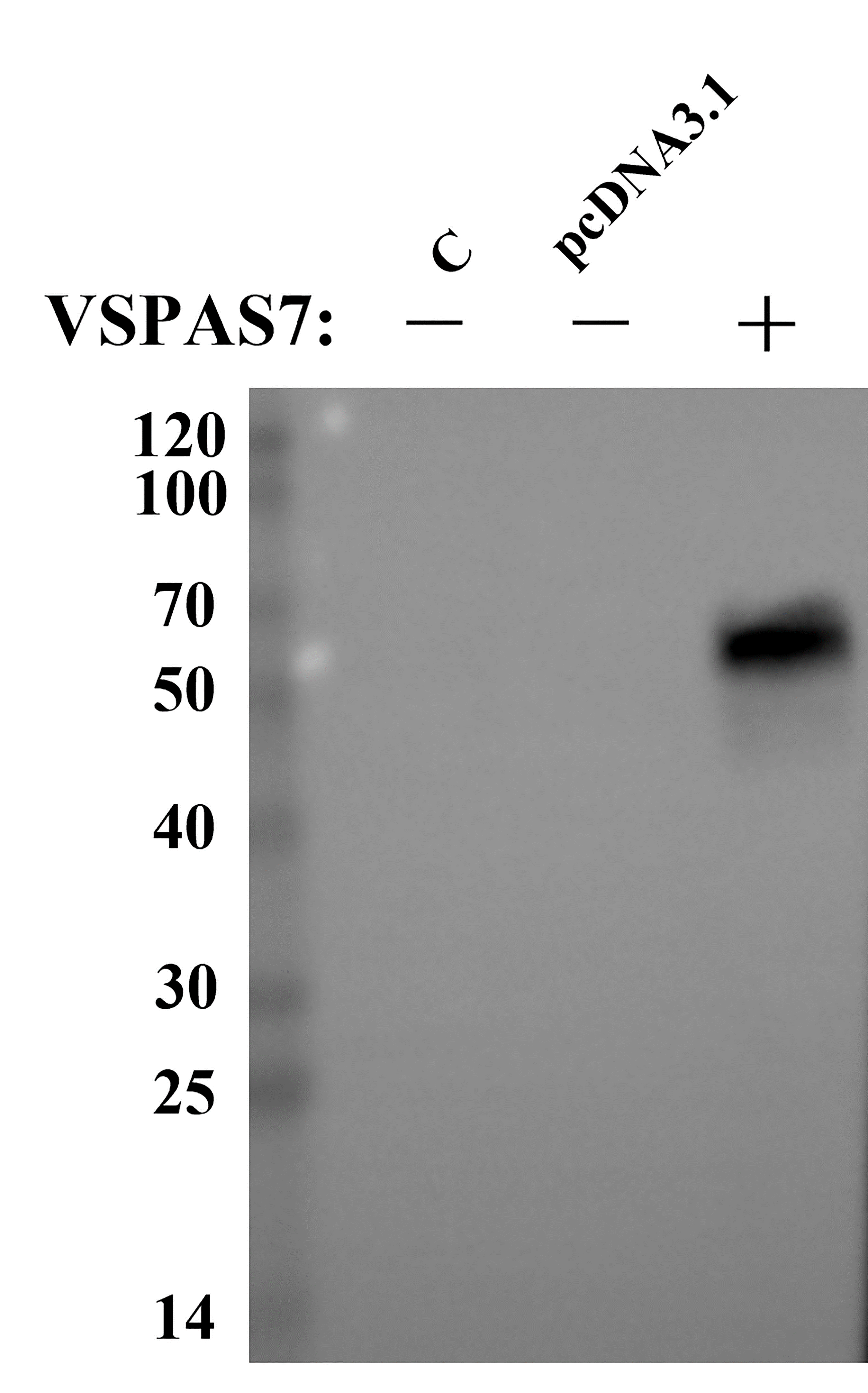


**Fig. S1 The expression level of VSPAS7.** Western blot of VSPAS7 expression were performed in mouse PMs transfected with the pcDNA3.1-*vspas7* vector at 48 h after transfection. C: Control.


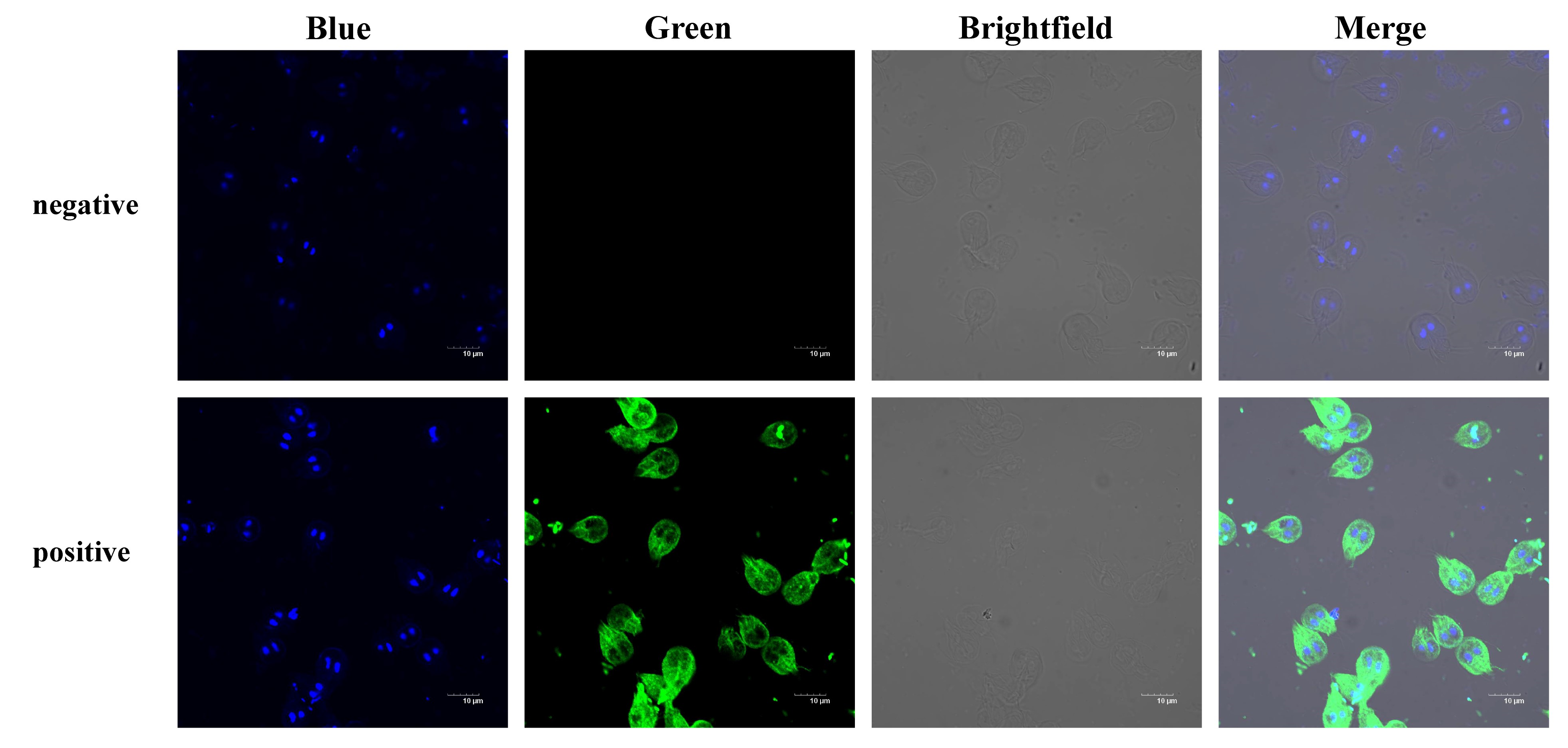


**Fig. S2 The localization of VSPAS7 in *Giardia*.** *Giardia* nucleus and VSPAS7 were detected by laser confocal microscopy. Blue: Hoechst 33342. Green: *Giardia* VSPAS7. Scale bar: 10 μm. negative: Rabbit-negative serum as primary antibody incubated with *Giardia*; positive: Rabbit anti-VSPAS7 protein antibody was used as primary antibody to incubate *Giardia*.
